# Surgical Treatment of Femoral Pseudarthrosis in a Child With Significant Rotational Deformity: A Case Report

**DOI:** 10.7759/cureus.96867

**Published:** 2025-11-14

**Authors:** Keisuke Yoshida, Yohei Kumabe, Keisuke Oe, Tomoaki Fukui, Ryosuke Kuroda

**Affiliations:** 1 Department of Orthopedic Surgery, Kobe University Graduate School of Medicine, Kobe, JPN

**Keywords:** diaphyseal femur fracture, femoral pseudarthrosis, one-stage correction, rotational deformity, wake-up test

## Abstract

Pediatric femoral pseudarthrosis is rare, and particularly in cases with significant rotational deformity, insights regarding treatment guidelines and the safety of surgical techniques are limited. In this report, we present a case of pediatric femoral pseudarthrosis that achieved good bone union and functional recovery through one-stage rotational correction combined with a wake-up test to avoid neurological complications. A 12-year-old boy, who had undergone surgical fixation for a right diaphyseal femur fracture at a previous hospital, was diagnosed with pseudarthrosis with 56° of external rotation deformity. At our institution, after freshening the pseudarthrosis site, we performed a one-stage rotational correction of 56° using a Hoffman-type external fixator while evaluating the nerve injury with a wake-up test. Neither intraoperative nor postoperative nerve impairment was observed, and synostosis was achieved at 13 postoperative weeks.

In the correction of rotational deformities, particularly in cases with large correction angles, there is a risk of nerve damage due to traction or compression of peripheral nerves. The wake-up test is a simple method for directly assessing nerve function, even under conditions with limitations on monitoring equipment, and it is useful for the early detection and prevention of neurological complications. In this case, a 56° rotational correction for pediatric femoral pseudarthrosis was performed in a one-stage procedure, and safe surgery was accomplished through intraoperative neurological assessment using the wake-up test. This suggests that the wake-up test can be a valuable auxiliary evaluation method even in situations where intraoperative nerve monitoring is limited.

## Introduction

Pseudarthrosis of the femoral diaphysis in children is rare, and there are no established treatment guidelines [1,2]. Typically, pediatric fractures have a high capacity for synostosis and often heal naturally. However, once pseudoarthrosis occurs, it can lead to long-term instability and functional impairment, significantly affecting the child's activities of daily living (ADL). Additionally, in cases where significant rotational deformity accompanies the pseudarthrosis, abnormal gait and a disruption of the load axis may result in patellar instability and pain in the hip/knee joints. Therefore, pseudarthrosis treatment and bone alignment correction are necessary. However, significant rotational correction cannot rule out the possibility of nerve damage due to extension caused by the movement of the nerves, so it is necessary to carefully consider the safe range and methods for modification. In tibial and femoral rotational correction surgeries, there are options for one-stage correction and gradual correction, each with reported advantages and issues [3]. This case involves a pediatric femoral pseudarthrosis with significant rotational deformity, for which a one-stage rotational correction was performed under intraoperative neuro-monitoring using the wake-up test in conjunction with pseudarthrosis surgery. This approach resulted in good bone union and functional recovery. This report describes the details of the surgical technique and postoperative course, discussing the risks and countermeasures of neurological complications in rotational correction surgery, the usefulness of the wake-up test, and a comparative evaluation of one-stage versus staged corrections based on the literature.

## Case presentation

Medical history

**Figure 1:**
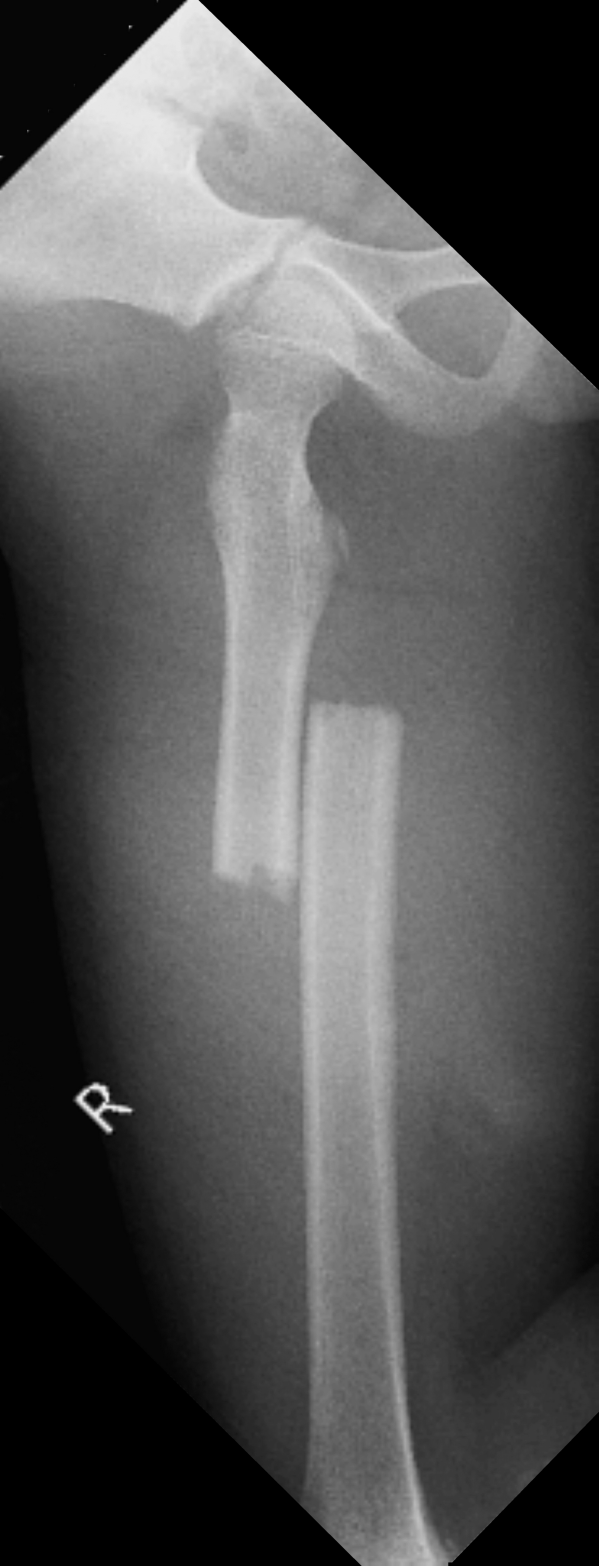
An anteroposterior radiograph of the right femur at the time of injury, showing a transverse mid-shaft fracture of the femoral diaphysis.

**Figure 2:**
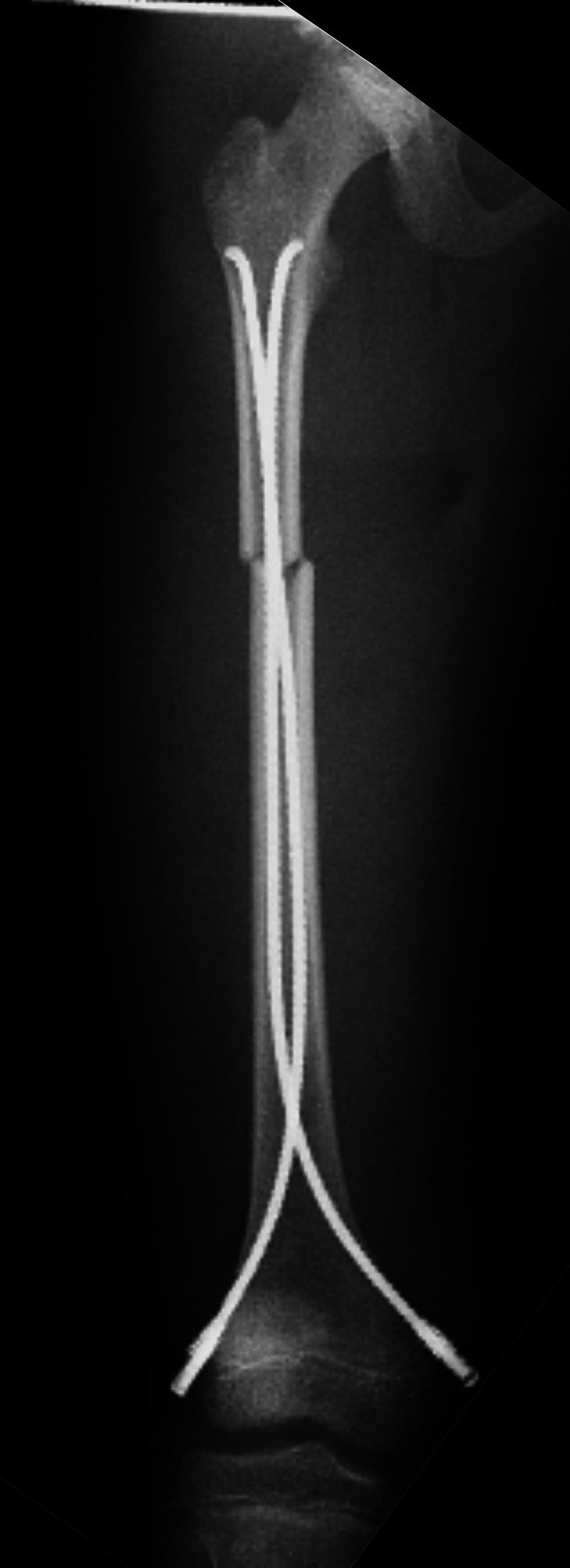
An anteroposterior radiograph of the right femur immediately after the initial surgery. It shows two intramedullary titanium elastic nails (TEN) in place across the fracture site.

A 12-year-old boy, 147 cm tall and weighing 36 kg, with no comorbidities, fell from a tree and sustained a transverse fracture of the right femoral shaft (Figure 1). An open reduction and internal fixation (Synthes TEN, φ4 mm) was performed by a previous physician (Figure 2).

From the day after the surgery, partial weight-bearing walking was permitted. During the course, external rotation deformity progressed, and five months postsurgery, there was no evidence of bone healing, leading to a diagnosis of right femoral pseudarthrosis accompanied by 56° of external rotation deformity. He was referred to our hospital for treatment. We consider that the cause was insufficient fixation strength and an inadequate non-weight-bearing period.

Present status

**Figure 3:**
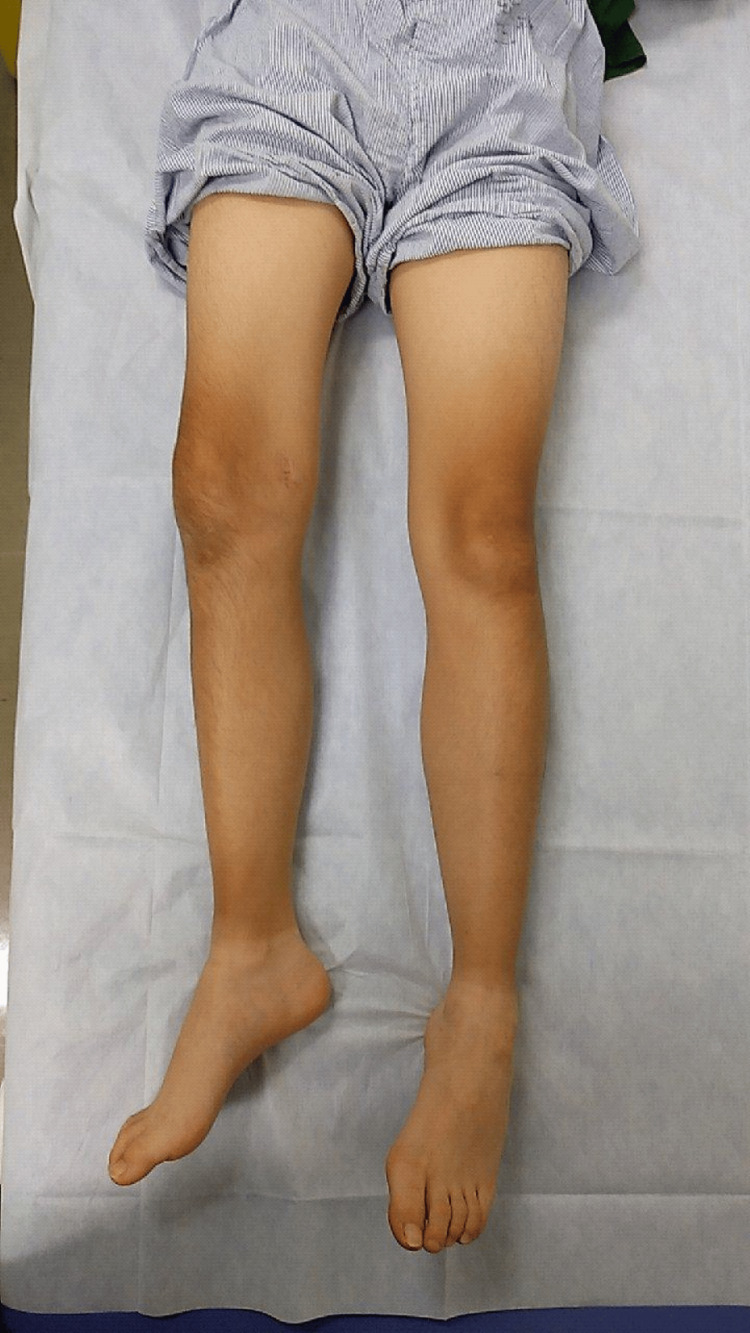
Clinical appearance of the right lower limb external rotation deformity before pseudarthrosis surgery. There is a marked external rotation deformity of the right distal femur (the right foot is pointing outward compared to the left).

**Figure 4:**
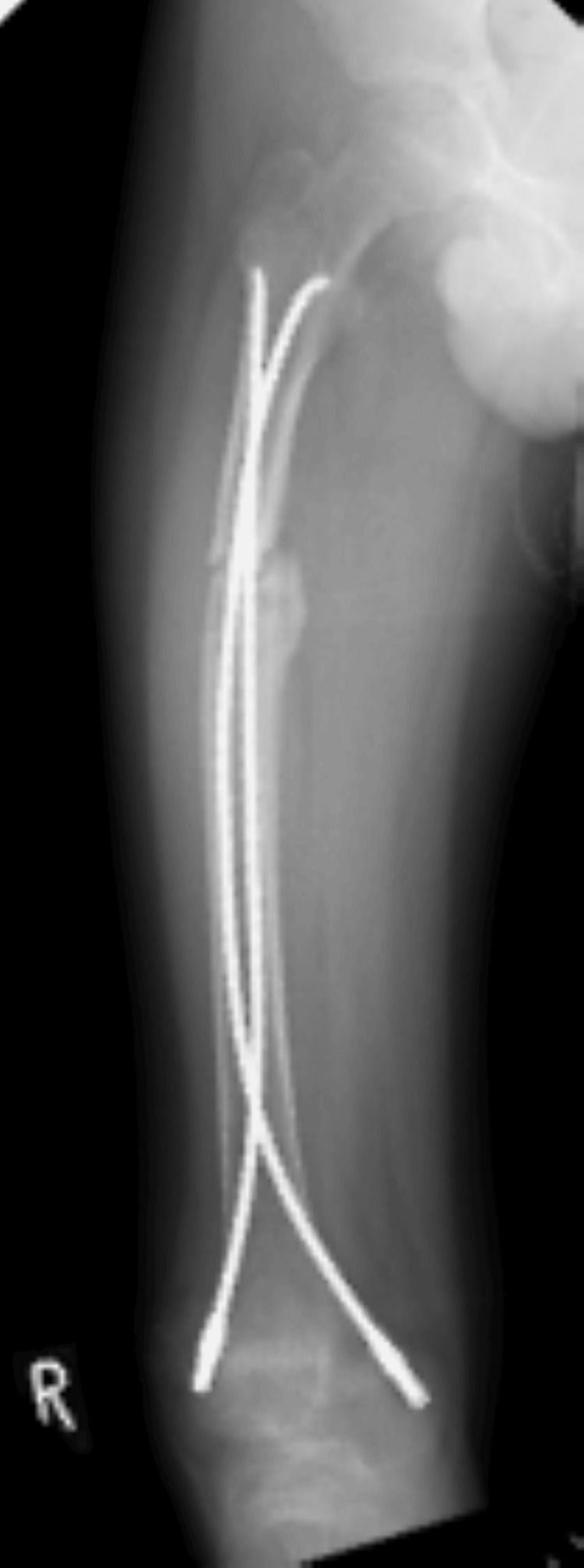
An anteroposterior radiograph of the right femur in a state of pseudarthrosis five months after injury. It shows a persistent non-union (pseudarthrosis) at the proximal third of the femoral shaft, with the flexible intramedullary nails still in situ.

**Figure 5:**
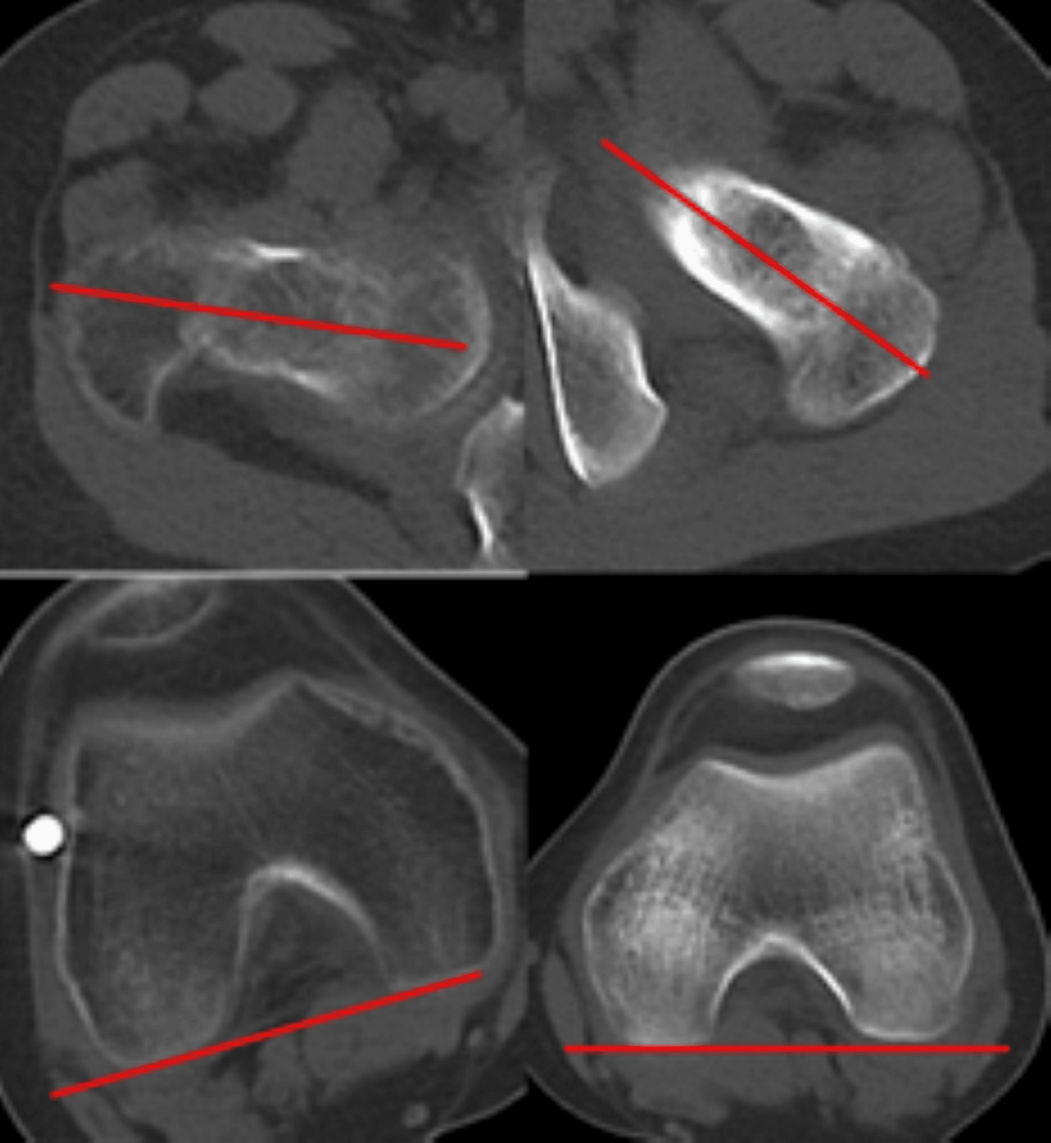
Preoperative CT scans of the right femur confirming the pseudoarthrosis and severe rotational malalignment. The distal fragment is externally rotated by 56° relative to the proximal fragment, consistent with the measured deformity.

Active motion of the right hip and knee is preserved. On inspection, the right lower limb shows an external rotation deformity above the right knee, and he walks with crutches (Figure 3). No leg length discrepancy was observed. He complained of pain during loading, and the range of motion in his joints was 0° extension and 100° flexion in the right hip joint, and -10° extension and 130° flexion in the right knee joint. Plain radiographs and CT scan findings revealed a non-union in the proximal one-third of the right femoral diaphysis, and significant external rotation deformity of the distal femur was confirmed (Figure 4). The anteversion angle was measured using CT scan as indicated by the red lines, which represent the femoral neck axis and the posterior condylar axis. These axes were used to quantitatively assess femoral rotation. The measured twist of the bone axis on the CT scan showed an external rotation position of 56° compared to the unaffected side (Figure 5).

Operative findings

**Figure 6:**
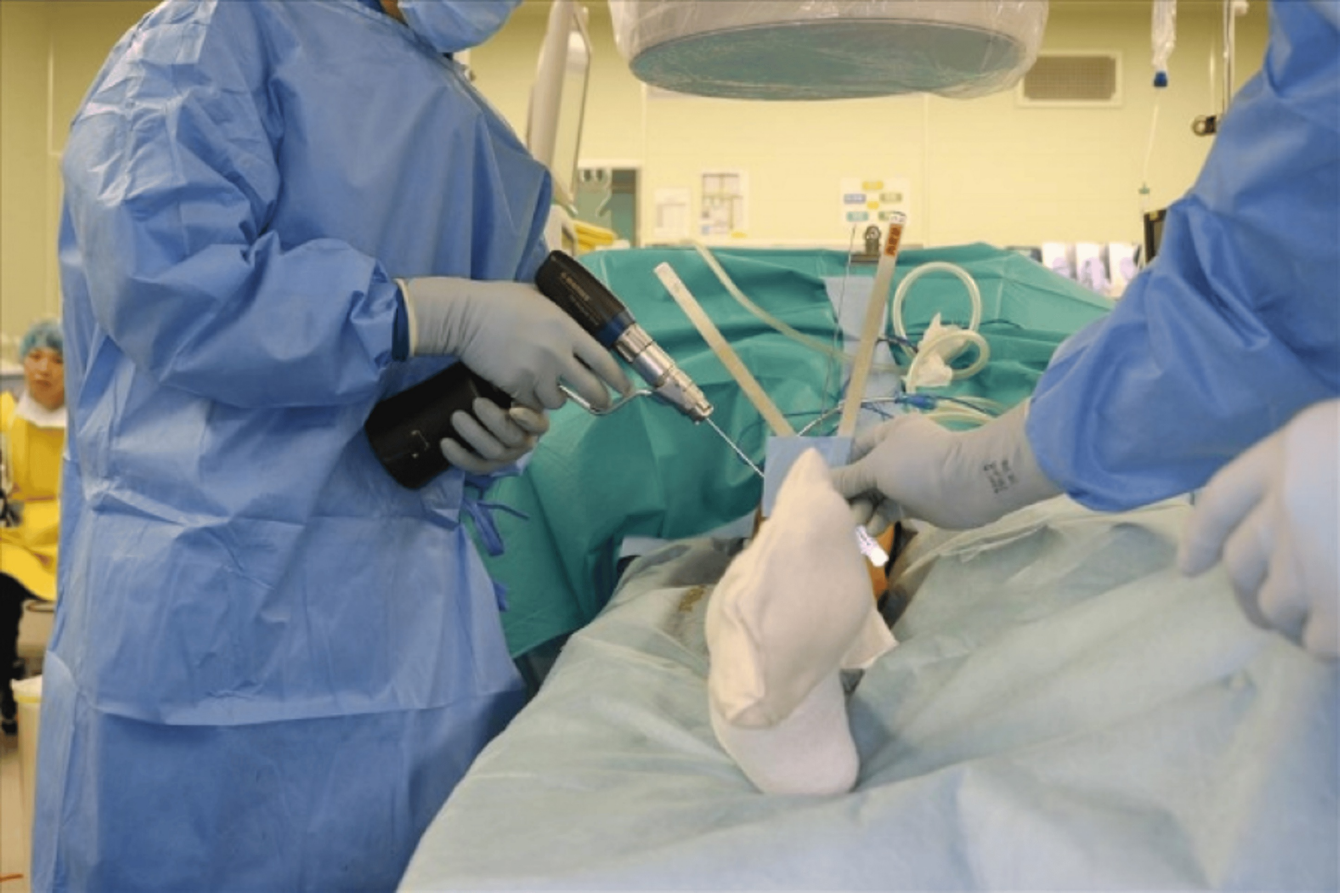
Intraoperative photograph showing placement of Kirschner wires (K-wires) in the proximal and distal femur at a 56° relative angle. These guide wires indicate the magnitude of the external rotation deformity and serve as references for the subsequent one-stage correction.

**Figure 7:**
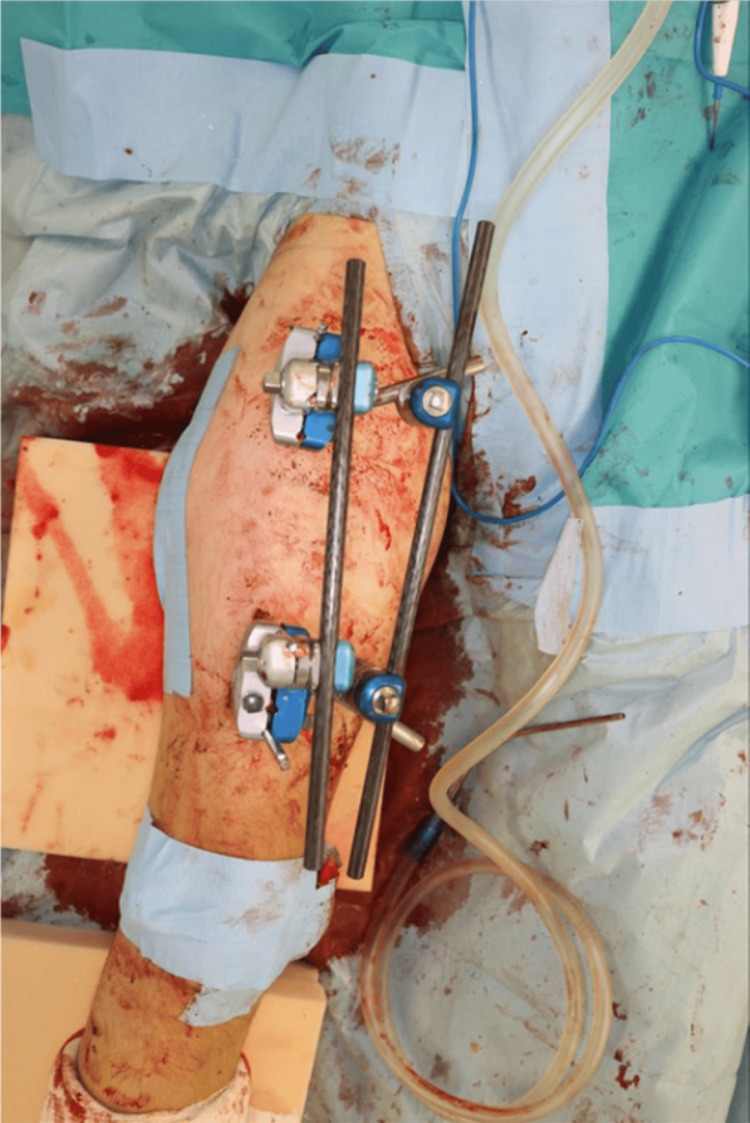
Intraoperative photograph showing application of a Hoffmann-type external fixator across the femur after removal of the intramedullary nails. The external fixator stabilizes the femoral fragments at the pseudarthrosis site prior to performing the one-stage rotational correction.

**Figure 8:**
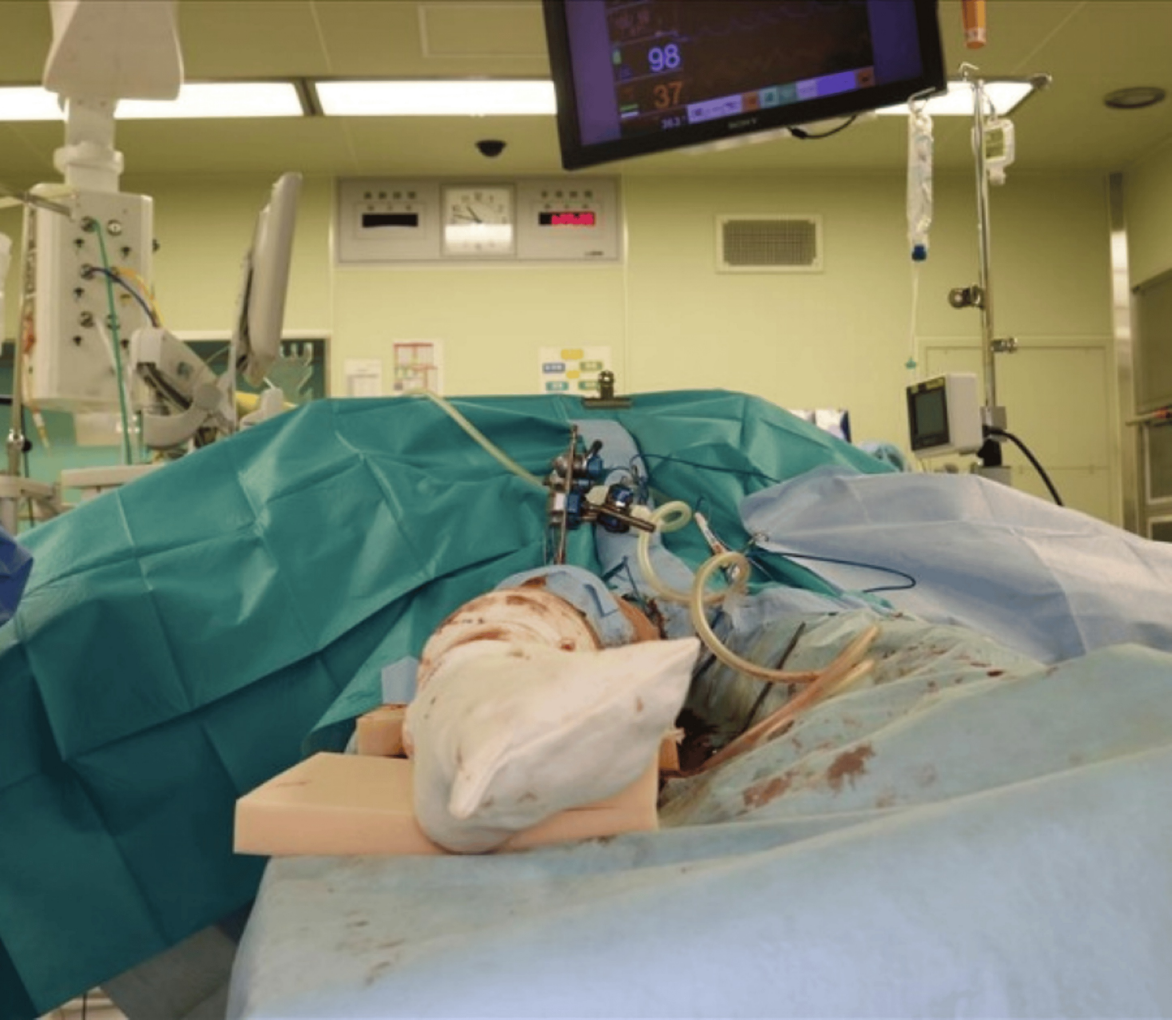
Intraoperative photograph during the one-stage rotational correction of the femur. The distal fragment has been rotated internally by 56° (using the K-wires as guides) to restore normal alignment with the proximal fragment.

Under general anesthesia, K-wires were placed at an external rotation angle of 56° for the proximal and distal bones as an indicator of the external rotation angle (Figure 6). The muscles were bluntly separated from the lateral aspect of the thigh to expose the shaft of the femur, and the pseudarthrosis site was scraped and freshened. The implant titanium elastic nail (TEN) was removed, and a Hoffman-type external fixator was installed, followed by a one-stage rotational correction using the pre-installed K-wires as a guide to maintain the reduction state (Figures 7, 8).

**Figure 9:**
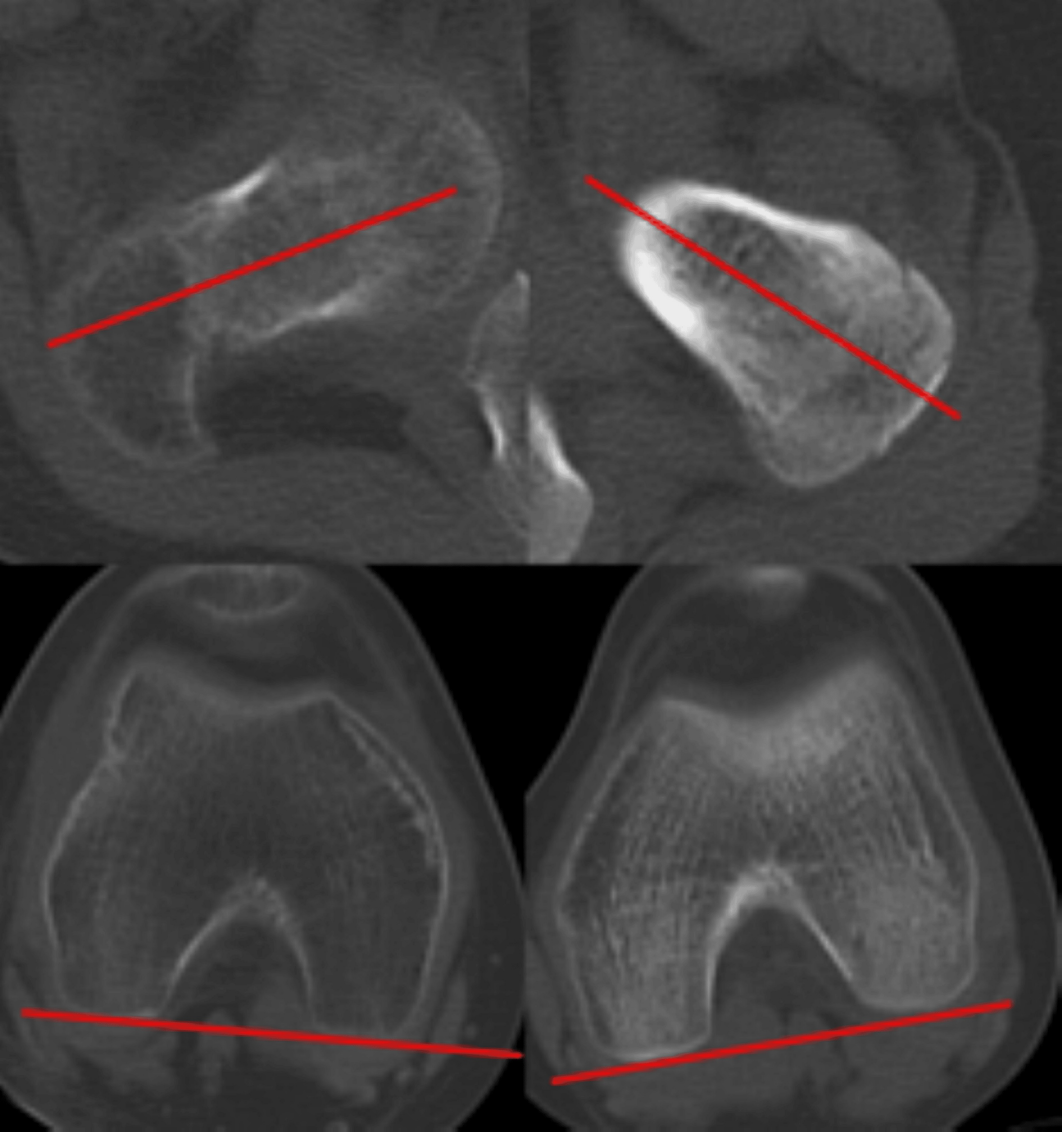
Postoperative CT scans of the right femur confirming complete correction of the rotational deformity. No residual external rotation misalignment is present, indicating successful 56° rotational realignment of the femoral shaft.

**Figure 10:**
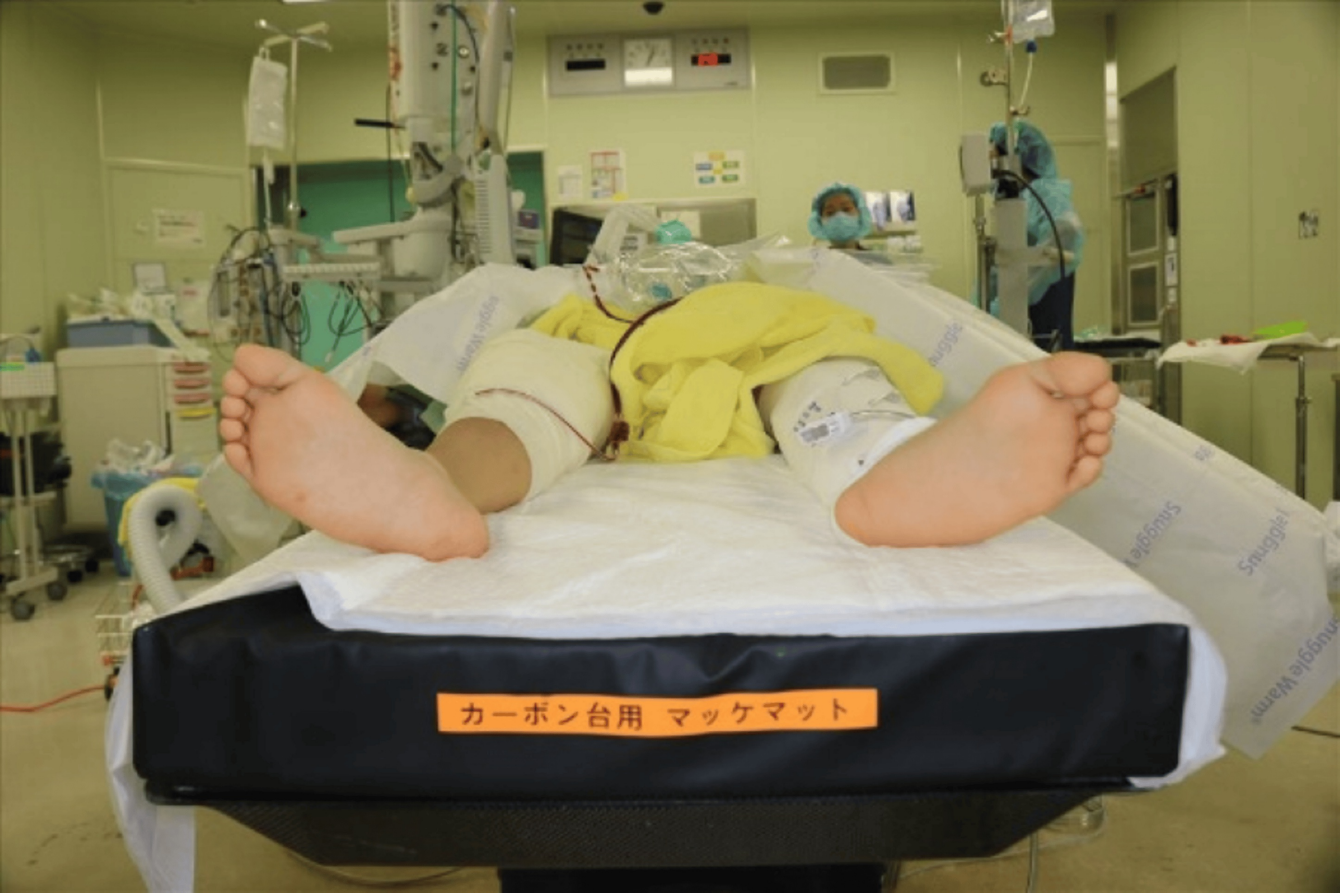
Clinical appearance of the right lower limb with resolution of external rotation deformity after pseudarthrosis surgery. The right knee and foot are now oriented forward, matching the left side, with no visible residual deformity.

**Figure 11:**
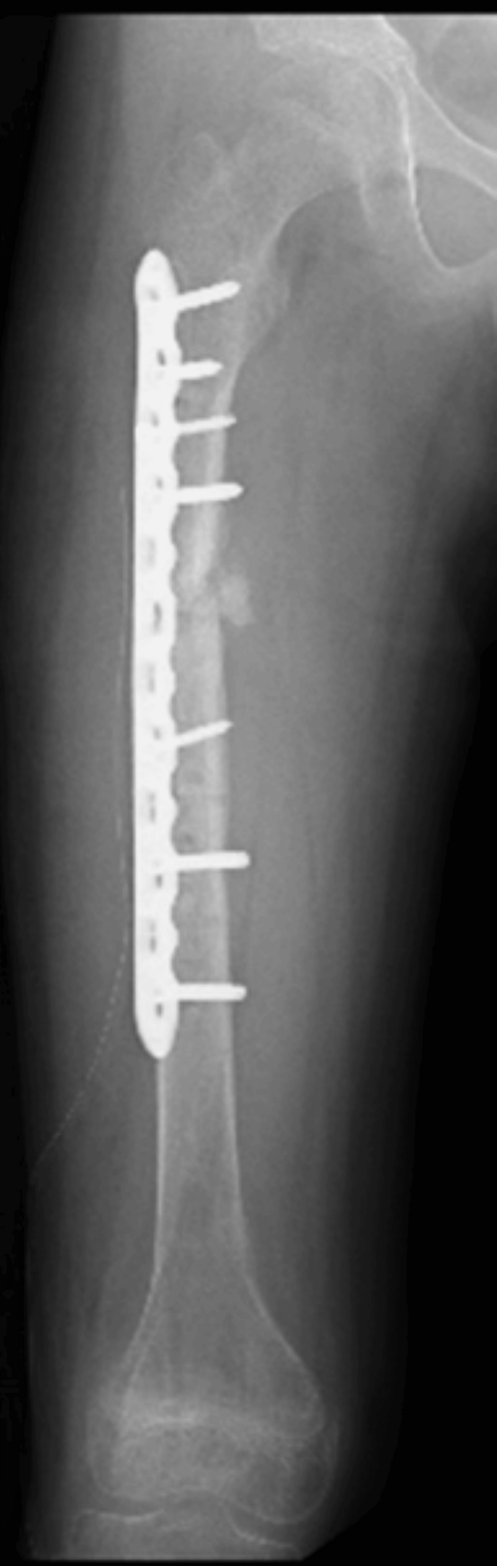
Postoperative anteroposterior radiograph of the right femur after the one-stage rotational correction and internal fixation. A lateral locking plate and screws securely bridge the former pseudarthrosis site, and the femur is in proper alignment with no remaining deformity.

A wake-up test was performed before internal fixation. The anesthesiologist was consulted, and the administration of intravenous anesthetic agents was temporarily suspended to reduce the level of consciousness, stimulating the patient through verbal cues. The patient voluntarily responded to simple verbal commands during awakening from anesthesia, showing automatic dorsiflexion and plantar flexion of the right ankle and toes. This confirmed that there were no neurological abnormalities during the rotational correction (such as the occurrence of common peroneal nerve paralysis or sciatic nerve paralysis). The wake-up test took about 15 minutes. With the rotational correction maintained, osteosynthesis was achieved using a 12-hole Synthes LCP broad plate with bicortical screws, and the external fixator was removed. After complete awakening from anesthesia, both the movement evaluation of the toes and foot and the sensory evaluation of the lower leg showed no findings suggestive of neurological impairment. Additionally, postoperative plain radiographs and CT scans confirmed the disappearance of the external rotational deformity (Figures 9-11).

Postoperative progress

**Figure 12:**
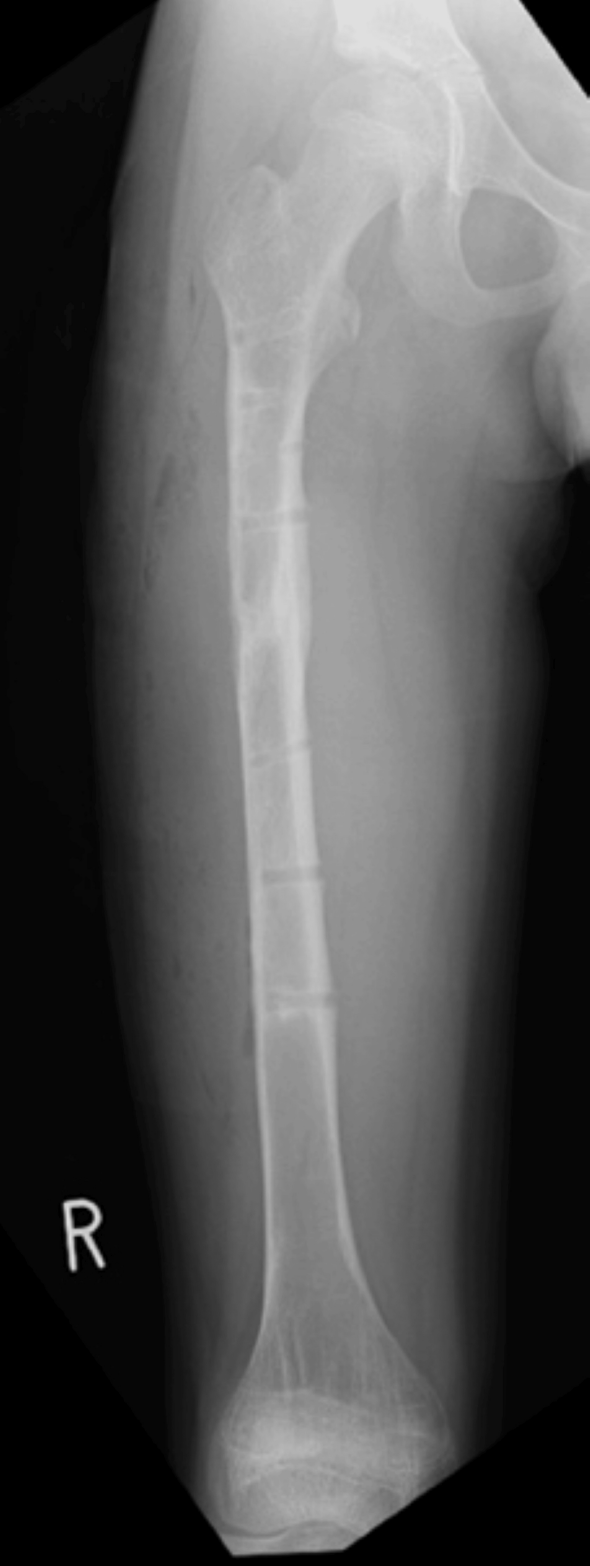
Follow-up anteroposterior radiograph of the right femur after hardware removal (eight months postsurgery). The femur shows solid bony union at the previous fracture site with normal anatomical alignment maintained.

Passive range of motion training for the hip and knee joints was initiated on the first postoperative day. After six weeks of non-weight-bearing, partial weight-bearing was initiated. Good bone union was observed in the plain radiographs obtained 13 weeks postoperatively, demonstrating a solid union, and full weight-bearing was allowed. The subsequent course was uneventful, and the plate was removed eight months postsurgery (Figure 12).

At one year, the patient had returned to sports. Throughout the follow-up period, no gait abnormalities, muscle weakness, re-fractures, or recurrence of deformity were observed. The range of motion of the right knee joint is +10° extension and 140° flexion. All diagnostic and assessment tools used in this study, including the wake-up test, are freely available for use without licensing restrictions.

## Discussion

Here, we report one instance in which significant rotational deformity correction and pseudarthrosis treatment for pediatric femoral pseudarthrosis were performed in one stage, successfully avoiding neurological complications through intraoperative neurological assessment using the wake-up test. Considering the background literature, we discuss the characteristics of this case and the key points of treatment.

In lower limb rotational correction, there is a possibility that peripheral nerves (especially the common peroneal nerve, femoral nerve, or sciatic nerve) may be elongated or compressed due to rapid changes in the positional relationship of bone fragments. Examples include common peroneal nerve palsy after tibial osteotomy, and femoral and sciatic nerve palsy after pelvic osteotomy around the hip joint (periacetabular osteotomy {PAO}) [4,5]. Also, past reports of adult femoral osteotomies have shown instances of transient sciatic nerve paralysis occurring postoperatively [6]. The pseudarthrosis site in this case is located in the central part of the femoral diaphysis, which is far from the sciatic nerve, so the risk of direct nerve injury is considered low. However, the probability of functional impairment due to twisting and elongation of the peripheral nerve trunk in the soft tissue, resulting from sudden changes in femur rotational angles, cannot be excluded. Therefore, in cases like this one with a large rotational correction angle in the femur, it is crucial to consider intraoperative nerve monitoring and nerve protection as necessary.

In this case, the wake-up test was used in conjunction with rotational correction as an immediate evaluation method for nerve tension. The wake-up test is a classical method devised for the early detection of spinal cord paralysis during spinal deformity correction surgery, first reported by Vauzelle et al. in 1973 [7]. Before the development of spinal monitoring technology, the only way to assess spinal and nerve function during surgery was to temporarily awaken the patient and check if they could voluntarily move their limbs. As the 1980s approached, neurophysiological monitoring techniques, such as transcranial evoked potentials (motor evoked potential {MEP}), were developed, and various electrophysiological methods for monitoring the brain, spinal cord, and peripheral nerves during surgery have been established in recent years [8]. Currently, due to advancements in intraoperative monitoring technology, the opportunities to perform the wake-up test have decreased; however, it remains a valuable auxiliary test in situations where monitoring devices cannot be adequately used, where the reliability of evoked potentials in children is low, or when unclear changes occur in monitoring findings [9]. There are reports that similar monitoring was conducted during or immediately after surgery for limbs with the possibility of nerve damage, aimed at evaluating nerve function [10]. At our facility, intraoperative MEP monitoring was difficult, so the wake-up test was adopted as a simple and direct method for evaluating nerve function. Fortunately, immediate confirmation of ankle dorsiflexion during intraoperative awakening was possible, and communication with the awakened patient was established, allowing for real-time assurance that there was no nerve compression related to the deformity correction. The wake-up test was completed quickly with the cooperation of the anesthesiologist, and surgery was resumed immediately after reintroducing anesthesia. The method of temporarily waking a patient during general anesthesia has been noted to carry risks associated with patient awakening during surgery (such as extubation or significant bleeding risks), as well as issues of patient anxiety and distress. However, in environments where monitoring means are limited, such as in this case, the wake-up test can be an effective means of avoiding neurological complications. To enhance its usefulness and safety, it is also important to communicate sufficiently with the patient before surgery and prepare them psychologically to prevent fear and panic upon awakening.

There are the following two methods for correcting bone deformities: "one-stage correction," which corrects the deformity to the target angle in a single instance during surgery, and "gradual correction," which uses devices like external fixators to gradually correct the deformity postoperatively [4]. The advantage of one-stage correction is that it can complete both deformity correction and bone healing in a single surgery, thereby reducing the patient's burden and shortening the time to bone union. However, reports have indicated that when the amount of correction is large, there is an increased risk of nerve damage, vascular injury, and compartment syndrome, which requires careful attention [11]. Gradual correction necessitates long-term external fixation management and carries risks of pin infections and joint contractures. Nevertheless, its advantage is that it allows for the gradual movement of the bone fragments over time, providing room for soft tissue extension and avoiding excessive tensile stress on nerves and vessels. Particularly in cases of severe deformities, with soft tissue contractures or scars, or in cases with poor soft tissue conditions due to prior surgeries or infections, a staged correction is preferred. It is advisable to assess the degree of deformity, age, and risk of complications comprehensively and to differentiate between one-stage and gradual correction accordingly. In this particular case, considering the goal of early healing of the pseudarthrosis and the patient's and family's wishes, we chose a one-stage correction. Ultimately, we achieved synostosis without postoperative nerve dysfunction, but we took maximum precautions for safety, such as directly confirming nerve function during surgery using a wake-up test.

## Conclusions

There is no established treatment method for rotational correction of pediatric pseudarthrosis cases involving significant rotational deformity. Intraoperative nerve damage is one of the complications that should be adequately considered, and whether to perform correction in a one-stage procedure should be a discussion point, depending on the individual case. In this case, a 56° rotational correction was safely performed in one stage without neurological complications, suggesting that this degree of correction may be feasible as a one-stage procedure in similar pediatric cases. Additionally, the wake-up test was found to be a simple and quick method for intraoperative nerve evaluation, which can aid in safely performing deformity correction surgery.
